# Dietary *Ganoderma lucidum* Modulates Liver and Adipose Tissue Responses in Western Diet-Fed C57BL/6J Mice

**DOI:** 10.3390/cimb48080797

**Published:** 2026-08-06

**Authors:** Catarina Castro-Ribeiro, Tiago Azevedo, Lillian Barros, Rita Silva-Reis, Mariana Gonçalves, Tiago Ferreira, João Ferreira, Rita Ferreira, Tânia Martins, Maria João Pires, Isabel Gaivão, Francisco Peixoto, Maria de Lurdes Pinto, Paula A. Oliveira

**Affiliations:** 1 Centre for the Research and Technology of Agro-Environmental and Biological Sciences (CITAB), Institute for Innovation, Capacity Building and Sustainability of Agri-food Production (Inov4Agro), University of Trás-os-Montes and Alto Douro (UTAD), 5000-801 Vila Real, Portugal; catarinaribeiro8f@gmail.com (C.C.-R.); ritareis96@hotmail.com (R.S.-R.); ma1ri2a3na4@gmail.com (M.G.); tiagoterras55@gmail.com (T.F.); joaommf93@hotmail.com (J.F.); taniam@utad.pt (T.M.); joaomp@utad.pt (M.J.P.); 2 CIMO, LA SusTEC, Instituto Politécnico de Bragança, Campus de Santa Apolónia, 5300-253 Bragança, Portugal; lillian@ipb.pt; 3 Animal and Veterinary Research Center (CECAV), Associate Laboratory for Animal and Veterinary Sciences (Al4AnimalS), University of Trás-os-Montes and Alto Douro (UTAD), 5000-801 Vila Real, Portugal; igaivao@utad.pt (I.G.); lpinto@utad.pt (M.d.L.P.); 4 LAQV-REQUIMTE, Department of Chemistry, University of Aveiro, 3810-193 Aveiro, Portugal; ritaferreira@ua.pt; 5 Research and Development Unit, Department of Human Genetics, National Institute of Health Doutor Ricardo Jorge, INSA I.P., 4000-055 Porto, Portugal; 6 Center for the Study of Animal Science (CECA), Associate Laboratory for Animal and Veterinary Sciences (Al4AnimalS), University of Porto, 4051-401 Porto, Portugal; 7 Chemistry Center-Vila Real (CQ-VRV), University of Trás-os-Montes and Alto Douro (UTAD), 5001-801 Vila Real, Portugal; fpeixoto@utad.pt; 8 RISE-Health—Health Research Network, Faculty of Medicine, University of Porto, 4200-319 Porto, Portugal

**Keywords:** genotoxicity, hepatic steatosis, mushroom, natural compounds, obesity, oxidative stress

## Abstract

Sustained consumption of Western-style diets (WDs), rich in fat and cholesterol, challenges metabolic homeostasis and results in organ-specific alterations, notably hepatic lipid accumulation and adipose tissue dysfunction. Medicinal mushrooms such as *Ganoderma lucidum* have gained attention for their biological activities; however, the extent to which nutritionally realistic dietary intake of *G. lucidum* modulates tissue-specific metabolic adaptation under obesogenic conditions remains poorly defined. This exploratory study aimed to evaluate how dietary supplementation with a *Ganoderma lucidum* extract (GLE) modulates metabolic and organ-specific responses in a WD-fed preclinical mouse model. Forty-seven male C57BL/6J mice were assigned to five groups receiving a control diet (CTR), a cholesterol-enriched WD, or the WD supplemented with increasing concentrations of GLE (0.7%, 1.4%, or 2.8%). Over thirteen weeks, body mass and food and water intake were monitored weekly. Afterwards, mice were euthanized, and tissue and blood samples were collected for further analysis. Fasting glucose was lower in WD+2.8% GLE than in CTR, although no GLE-supplemented group differed significantly from WD and glucose tolerance was unchanged. All GLE-supplemented groups had a lower hepatic genetic damage index than WD. WD+1.4% GLE showed a higher frequency of localized periportal hepatic vacuolar changes than WD, whereas WD+2.8% GLE had lower relative liver weight. GLE supplementation was also associated with depot-specific variation in the occurrence of multilocular adipocytes with a beige-like appearance, without significant changes in final body mass or adiposity. In this preclinical model, subchronic dietary GLE was associated with selected tissue-specific differences, most consistently lower hepatic DNA damage and depot-specific variation in multilocular adipocyte morphology, without consistent systemic metabolic improvements.

## 1. Introduction

Western diet (WD) consumption reflects a dietary pattern characterized by frequent consumption of energy-dense, highly processed foods that are generally inexpensive, palatable, and convenient [1]. Originally associated with high-income countries, this dietary pattern has become increasingly prevalent worldwide, including in low- and middle-income regions, as a consequence of urbanization, globalization of food systems, and lifestyle changes that favor processed foods over traditional diets [2]. The high content of saturated fats, refined carbohydrates, and cholesterol in WDs imposes continued metabolic stress that disrupts physiological homeostasis [3]. Prolonged exposure promotes excess lipid availability [4], ectopic fat deposition [5], and low-grade inflammation [6], contributing to systemic metabolic dysregulation and increasing the risk of subchronic metabolic disorders, including obesity, type 2 diabetes, dyslipidemia, and non-alcoholic fatty liver disease [7,8,9,10], being also associated with increased mortality [11].

Importantly, the biological impact of WDs extends beyond body weight gain and involves organ-specific alterations in tissues central to metabolic regulation, particularly adipose tissue and the liver [12]. Under conditions of nutritional excess, adipose tissue expands through adipocyte hypertrophy and hyperplasia and undergoes functional remodeling that influences lipid metabolism, inflammatory response, and endocrine signaling [13]. In parallel, the liver, a crucial organ in lipid and glucose homeostasis, is highly vulnerable to WD-induced metabolic stress [14]. Increased fatty acid influx, altered de novo lipogenesis, and impaired lipid export contribute to hepatic lipid accumulation [15], which may compromise cellular integrity and genomic stability. These tissue adaptations occur early during metabolic imbalance and may precede explicit metabolic disease, highlighting the relevance of evaluating organ-specific responses to dietary stress.

Dietary strategies capable of modulating these early physiological adaptations are therefore of increasing interest, particularly when based on bioactive compounds that can be integrated into habitual dietary patterns rather than administered as pharmacological interventions [16]. In this context, naturally occurring food-derived bioactives have attracted attention due to their biological activity, safety profiles and potential to enhance metabolic resilience [17,18,19]. Edible and medicinal mushrooms represent complex food matrices rich in polysaccharides, sterols, phenolic compounds, and triterpenoids, many of which have been reported to influence metabolic and inflammatory pathways in experimental models [20,21].

Among these, *Ganoderma lucidum* has a long history of dietary and traditional use and has been extensively chemically characterized. Its major bioactive classes, particularly polysaccharides and triterpenoids, have been associated with antioxidant, anti-inflammatory, and hepatoprotective activities [22,23,24]. However, despite growing interest in G. lucidum extracts (GLEs), many experimental studies have relied on acute or pharmacological administration protocols, frequently involving purified fractions or bolus dosing that does not reflect sustained dietary exposure [25,26]. Conversely, the relatively limited number of studies investigating dietary incorporation of *G. lucidum* have often focused primarily on systemic metabolic outcomes such as body weight or glucose tolerance, reporting modest or inconsistent effects [27]. Importantly, these studies have generally not examined early tissue-level responses in organs central to metabolic regulation, nor have they evaluated parameters related to hepatic genomic stability or adipose tissue morphological remodeling, which may precede overt metabolic alterations. As a result, the potential influence of sustained dietary GLE intake on organ-specific adaptations to obesogenic dietary stress remains insufficiently characterized.

Against this background, the present exploratory study aimed to evaluate whether subchronic dietary supplementation with nutritionally relevant concentrations of a GLE modulates metabolic, hepatic, and adipose tissue responses to WDs in male C57BL/6J mice, with particular attention to glycemic control, liver integrity, adipose tissue morphology, and redox status.

## 2. Materials and Methods

### 2.1. Preparation and Characterization of the Ganoderma lucidum Extract

The *G. lucidum* extract was prepared according to the procedure described by Taofiq et al. [28]. Briefly, dried fruiting bodies obtained from Bioreishi—Agricultura Biológica, Lda, Portugal (Batalha, Portugal), were powdered and extracted using a Soxhlet apparatus with ethanol as the solvent. After extraction, ethanol was completely removed under reduced pressure, minimizing the likelihood of residual solvent and excluding any confounding effects related to alcohol intake, and the dried extract was stored at 4 °C until use. For dietary supplementation, the dried extract was incorporated directly into the diet at the desired concentrations (0.7–2.8%, *w*/*w*) during feed preparation.

The chemical profile of the GLE used in the present study was previously characterized by HPLC-DAD-ESI/MS^n^ [28]. The GLE contained 455 ± 1 mg of total triterpenoids and 6.30 ± 0.01 mg of total phenolic acids per gram of extract. The main quantified triterpenoids (expressed as relative abundance within the quantified chemical profile) included ganoderic acid H (8.91%), ganoderic acid C2 (8.39%), ganoderic acid A (7.97%), ganoderic acid C6 (6.31%), ganoderic acid η (5.51%), ganoderic acid B (5.20%), ganoderic acid F (3.89%), ganoderic acid G (3.65%), ganoderic acid AM1 (3.43%), 3,7,15-trihydroxy-4-(hydroxymethyl)-11,23-dioxo-lanost-8-en-26-oic acid (3.08%), 20-hydroxyganoderic acid AM1 (2.47%), ganoderic acid D (2.45%), ganoderic acid E (2.34%), ganoderenic acid B (2.31%), and ganoderenic acid D (2.23%). Several additional ganoderic acid and related triterpenoid derivatives were also identified at lower individual abundances. The phenolic acid fraction (similarly expressed as relative abundance) comprised *p*-hydroxybenzoic acid (0.65%), protocatechuic acid (0.39%), and syringic acid (0.33%).

### 2.2. Animals and Ethical Approval

The animal experiment was approved by the local Animal Welfare and Ethical Review Body (ORBEA) at the University of Trás-os-Montes and Alto Douro (UTAD) and the national competent authority Direção-Geral de Alimentação e Veterinária (DGAV, Lisbon, Portugal; license no. 004116; approved on 16 March 2023). This study was conducted in accordance with the Portuguese Law (Law Decree no. 113/2013) and the European Directive 2010/63/EU on the protection of animals used for scientific purposes.

Forty-seven male C57BL/6J mice aged between 49 and 55 days were acquired from Charles River Laboratories (Saint-Germain-Nuelles, France). The number of animals was determined based on previous exploratory dietary intervention studies using C57BL/6J mice [29,30] and to ensure adequate statistical power for the planned analyses, balancing the ability to detect physiologically relevant differences with ethical considerations related to animal use. Mice were kept at the University of Trás-os-Montes and Alto Douro’s animal facilities under controlled temperature (20 ± 2 °C), relative humidity (50 ± 10%) and photoperiod (12 h/12 h light–dark cycle). Mice were housed in open polycarbonate cages with corn-cob bedding and environmental enrichment (e.g., cardboard rolls, wooden blocks and paper) to allow normal teeth wear due to the diet’s soft texture. The animals were left to acclimate with unrestricted access to standard food (Diet Standard 4RF21 Certificate, Mucedola, Milan, Italy) and tap water, and bedding was changed weekly.

### 2.3. Experimental Design

After the acclimation period, the animals were randomly assigned to five groups (two cages per group) and were fasted for 12 h before the start of exposure to the new diets. Randomization was generated in Microsoft Excel (Microsoft, Redmond, WA, USA) using the random number function. Due to the absence of nuisance factors, the experimental design did not require the implementation of randomized block designs, but the order of experimental groups was mixed during the procedures. The diets were the control diet (CTR; Western 1635 control; SAFE^®^, Augy, France) and the Western diet with 0.20% cholesterol (WD; Western 1635 + 0.20% cholesterol; SAFE^®^, Augy, France). Increasing concentrations of a *G. lucidum* extract (0.7%, 1.4%, and 2.8%) were incorporated into the Western diet containing cholesterol, resulting in five experimental groups: Western diet control (CTR, *n* = 8); Western diet+0.2% cholesterol (WD, *n* = 9); WD+0.7% GLE (*n* = 9); WD+1.4% GLE (*n* = 10); and WD+2.8% GLE (*n* = 11). These concentrations were defined based on previous studies and, using standard allometric scaling and typical mouse intake [31], designed to correspond approximately to intakes that could realistically be incorporated into daily human diets, on the order of a few grams to the low tens of grams of extract per day (corresponding to approximately 4, 8 and 16 g of GLE per day).

The experimental study had a duration of 13 weeks. The animals were weighed weekly and their food and water consumption recorded using a precision scale (KERN^®^ PLT 6200-2A, KERN & SOHN GmbH, Balingen, Germany). During this time, animal welfare was checked and scored against a predefined humane endpoint table to ensure animal welfare [32]. Throughout the study, analyses were performed using coded samples.

#### 2.3.1. Assessment of Glucose Tolerance

The glucose tolerance test was conducted in weeks 7 and 13. Mice were fasted overnight and subsequently administered a glucose solution (2 g kg^−1^) via intraperitoneal injection, in accordance with established protocols [33]. Blood samples were collected immediately prior to the injection, and at 30, 60, and 120 min post-injection via tail puncture using an OGCare glucometer (BSI Diagnostics, Arezzo, Italy). Glycemic responses were expressed as glucose concentration over time and area under the curve (AUC).

#### 2.3.2. Tissue Collection and Euthanasia

At the end of the experimental period, mice were fasted for 24 h and euthanized by intraperitoneal overdose of ketamine (75 mg kg^−1^; Clorketam 1000, Vétoquinol, Barcarena, Portugal) and xylazine (45 mg kg^−1^; Rompun^®^ 2%, Bayer Healthcare S.A., Kiel, Germany). After confirmation of deep anesthesia and absence of reflex responses, blood was collected by cardiac puncture for the determination of fasting blood glucose concentrations using an OG Care glucometer (BSI Diagnostics, Arezzo, Italy).

The naso-anal length of each animal was measured using a ruler to determine the Lee index, following the formula [34]:

(1) $$Lee\;index=\frac{\sqrt[{3}]{Final\;body\;mass\;(g)}}{Naso-anal\;length\;(cm)}\times 100$$

At necropsy, the liver, adipose tissue depots (anterior subcutaneous, posterior subcutaneous and abdomino-pelvic), kidneys, heart, lungs, spleen, and thymus were collected, macroscopically assessed, and weighed. For liver analyses, samples were collected from the same liver lobe for each analysis across all animals. Samples for histology were fixed in 10% neutral buffered formalin, and samples for genotoxicity (comet assay) and oxidative stress analyses were stored at −80 °C. Adipose tissue depots and kidneys were fixed in 10% neutral buffered formalin for histological evaluation. The remaining organs were collected for morphometric assessment only.

The relative weight of each organ and adipose tissue depot was determined using the following formula:

(2) $$Relative\;organ\;weight=\frac{Organ/adipose\;depot\;mass\;(g)}{Final\;animal\;body\;mass\;(g)}$$

Total body fat was recorded as the sum of the masses of fat taken from the abdomino-pelvic, anterior subcutaneous and posterior subcutaneous depots. The adiposity index was calculated using the following formula [35]:

(3) $$Adiposity\;index\;(\%)=\frac{Total\;body\;fat\;deposits\;(g)}{Final\;animal\;body\;mass\;(g)}\times 100$$

### 2.4. Histological Evaluation of Liver, Adipose Tissue, and Kidney

Tissue samples fixed in 10% buffered formaldehyde were subsequently sectioned and processed following standard protocols for paraffin embedding. For histopathological analysis, microtomy was performed and 3 µm thick sections were stained with hematoxylin–eosin (H&E) for visualization by a blinded pathologist.

Liver histological findings were categorized into hydropic, microvacuolar, and/or macrovacuolar changes; these lesions were further classified based on their distribution (diffuse or localized). Hydropic change was defined as hepatocellular swelling with pale or rarefied cytoplasm, consistent with altered cellular water balance. Microvacuolar change comprised multiple small cytoplasmic vacuoles without nuclear displacement, whereas macrovacuolar change comprised one or more larger vacuoles that displaced the nucleus toward the cell periphery. The presence or absence of focal or necrotic hepatitis was also evaluated.

Adipose tissue from each depot was classified morphologically as white adipose tissue (WAT), multilocular adipocytes (MLAs) with a beige-like appearance, or WAT containing MLAs. This descriptive classification was based exclusively on H&E morphology and was not intended to establish a thermogenic beige or brown adipocyte identity.

Renal lesions were identified as chronic interstitial nephritis and chronic pyelonephritis. The presence of inflammatory cells in relation to arcuate arteries was also recorded.

### 2.5. Hepatic DNA Damage

The alkaline comet assay was performed as described by Collins et al. [36] to quantify single- and double-strand breaks in DNA. Whole-tissue liver cell suspensions were prepared in accordance with comet assay protocols previously applied to assess hepatic genomic integrity [37]. Thawed liver tissue from every animal was placed in PBS, cut to obtain a cell suspension, and centrifuged (200 *g*, 4 °C, 5 min). The pellet was resuspended in PBS and mixed with 140 µL of low-melting-point agarose (0.8–1% in PBS; A9414, Sigma-Aldrich, Saint Louis, MO, USA). Three 6 µL drops were placed on pre-coated (1% normal-melting-point agarose; A4718, Sigma-Aldrich) slides. Each slide contained samples from four animals loaded in triplicate mini-gels (12 mini-gels per slide). Slides were then cooled at 4 °C for 5 min. Slides were incubated in lysis buffer (2.5 M NaCl, 0.1 M EDTA, 0.01 M Tris, 1% Triton X-100, pH 10) at 4 °C for at least 1 h, then immersed in electrophoresis buffer (0.3 M NaOH and 1 mM EDTA, pH 12.6) for 30 min at 4 °C. Electrophoresis was conducted at 25 V, 300 mA (0.8 V/cm) for 25 min. Slides were rinsed with PBS and distilled water, fixed in 96% ethanol (10 min), and air-dried.

To visualize the cells, all the slides were stained (35 μL/slide) with 4′,6-diamidino-2-phenylindol (1 μg mL^−1^ in distilled H_2_O), and the observation was made using an epifluorescence microscope (BX41 Olympus, Hamburg, Germany) at 400× magnification. Cells were scored from 0 (no tail) to 4 (maximum damage) [36], and 100 comets were analyzed per mini-gel. DNA damage was expressed as a genetic damage index (GDI) on an arbitrary scale of 0–400, calculated using the following formula:

(4) GDI (AU)=∑% nucleoids class i×i

### 2.6. Hepatic Oxidative Stress

Liver samples were homogenized (1:10, *w*/*v*) in cold Tris-HCl buffer (pH 7.4) using a Potter–Elvehjem homogenizer coupled with a stirrer (Eurostar Power-B, IKA^®^ Werke, Staufen, Germany, 300 rpm). Homogenates were sonicated on ice (Vibra-Cell™ VCX 130, Sonics & Materials, Inc, Newtown, CT, USA; 3 × 20 s, with 20 s pauses) and centrifuged twice at 4000× *g* and once at 14,000× *g* for 10 min at 4 °C (Sigma 2-16K centrifuge, Sigma Laborzentrifugen GmbH, Osterode am Harz, Germany). Supernatants were collected and stored for enzymatic assays.

Catalase (CAT) (EC 1.11.1.6) activity was measured using a Clark-type oxygen electrode [38], by monitoring O_2_ formation from H_2_O_2_ decomposition in phosphate buffer (50 mM KH_2_PO_4_, pH 7.0). The reaction (1 mL) was initiated by adding 5 μL of sample to a pre-incubated (30 °C) buffer containing diluted H_2_O_2_ (10 μL, 8.82 M). O_2_ evolution was monitored for 2 min and expressed as mmol H_2_O_2_ min^−1^ mg^−1^ protein. Glutathione reductase (GR) (EC 1.6.4.2) activity was determined spectrophotometrically by monitoring NADPH oxidation at 340 nm [39]. The reaction mixture (2 mL) included phosphate buffer (100 mM KH_2_PO_4_, 0.5 mM EDTA, pH 7.4) and 40 μL of sample, and was incubated at 30 °C for 2 min. Subsequently, 20 μL NADPH (10 mM) and 20 μL GSSG (100 mM) were added. Absorbance was recorded over 3 min, and activity expressed as μM NADPH oxidized min^−1^ mg^−1^ protein (ε = 6.22 × 10^3^ M^−1^ cm^−1^). Protein content, used for enzyme activity normalization, was determined by the Biuret method using bovine serum albumin as a standard [40].

### 2.7. Statistical Analysis

Statistical analysis was carried out using GraphPad Prism version 10.4.1 (GraphPad Software, Inc., San Diego, CA, USA), and statistically significant differences were considered when *p* < 0.05. Data were analyzed for normality by the Shapiro–Wilk normality test and for homogeneity of variances using Levene’s test. A one-way analysis of variance (ANOVA) statistical analysis was performed, followed by Tukey’s multiple comparison test. Glucose-tolerance AUC values obtained at weeks 7 and 13 were analyzed separately using one-way ANOVA, as each test was considered a predefined, time-specific assessment. No formal comparison between weeks or analysis of within-animal temporal trajectories was performed. Data are expressed as mean ± standard error of the mean (SEM), except for the histological data, which are expressed in numbers of animals and percentages and were analyzed using the Chi-square test.

## 3. Results

During the experimental period, no mortality was recorded in consequence of GLE inclusion in the diet. The animals were monitored daily according to the predefined humane endpoint table, and none reached the criteria for euthanasia or exhibited any notable treatment-related behavioral changes.

### 3.1. Dietary Intake and Mean Body Mass

Food and water intake did not differ significantly between experimental groups at any time point during the intervention (Table 1). Minor temporal alterations in food and water consumption were observed within some groups; however, no consistent pattern attributable to GLE supplementation was detected.

Initial and final body mass, the Lee index, and the adiposity index for all experimental groups are presented in Table 2. All groups exhibited an increase in body mass over the experimental period. Mice in the CTR group showed the greatest overall increase in body mass, followed by the WD group. Animals receiving GLE supplementation also gained body mass, although to a lesser extent than Western diet controls. The Lee and the adiposity indices were generally comparable among WD-fed groups and, while both showed no statistically significant differences, the adiposity index exhibited a slight increase with increasing GLE concentration (*p* > 0.05).

### 3.2. Glycemic Homeostasis

Glycemic responses to intraperitoneal glucose administration, expressed as the AUC, are summarized in Table 3. At week 7, AUC values were comparable among all experimental groups (*p* > 0.05). Similarly, no statistically significant differences between groups were detected at week 13, indicating that GLE supplementation did not significantly affect glucose tolerance at either assessment (*p* > 0.05).

In contrast, fasting blood glucose concentrations measured at sacrifice differed between groups (Table 3). Fasting glucose was significantly lower in WD+2.8% GLE than in CTR; however, none of the GLE-supplemented groups differed significantly from WD (*p* < 0.05).

### 3.3. Relative Organ and Adipose Tissue Weight

Relative organ weights are presented in Table 4. Relative heart weight was higher in the WD+1.4% GLE group compared with the WD+2.8% GLE group (*p* < 0.05). A similar pattern was observed for lung weight, with the WD+2.8% GLE group exhibiting the lowest relative lung weight, significantly lower than all other groups (*p* < 0.05). Relative liver weight was significantly lower in WD+2.8% GLE than in WD (*p* < 0.05); however, neither group differed significantly from CTR.

Relative adipose tissue mass from individual depots is shown in Table 5. No significant differences were observed between groups in the relative mass of abdomino-pelvic or anterior subcutaneous adipose tissue. In contrast, posterior subcutaneous adipose tissue mass differed between groups, with the WD group showing a significantly lower relative mass compared with the WD+2.8% GLE group, which exhibited the highest value (*p* < 0.05).

### 3.4. Hepatic Tissue Alterations

**Figure 1:**
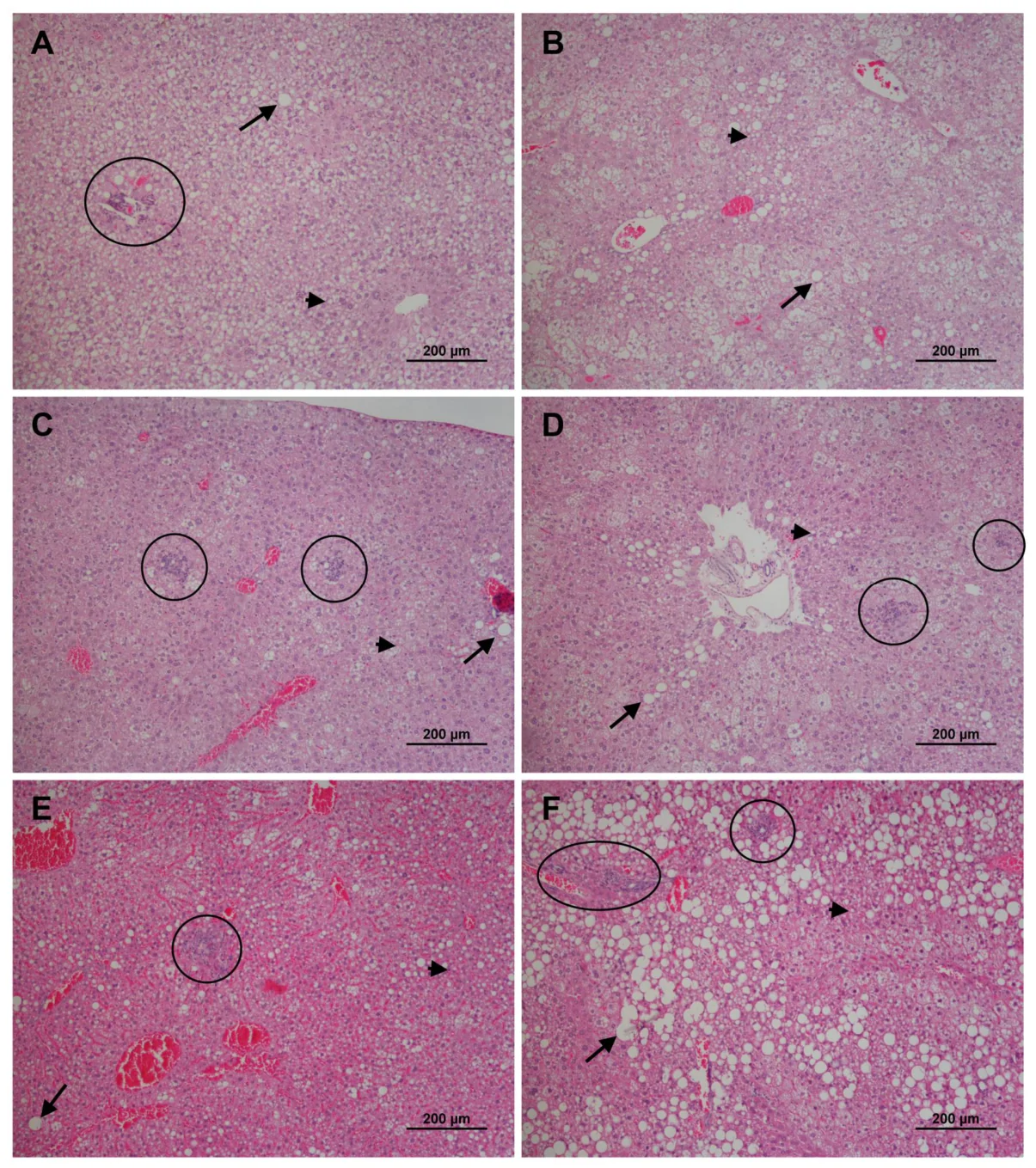
Representative histological micrographs of the hepatocellular changes found in the liver: micro- (arrowhead) and macrovacuolar (arrow) changes with diffuse (**A**–**C**,**E**,**F**) and periportal (**D**) distribution in the liver parenchyma. Chronic multifocal hepatitis, highlighted by black circles (**A**,**C**–**F**). Images illustrate the range of lesions identified during histopathological evaluation and not specific experimental groups. Quantitative data are presented in Table 6.

Histological evaluation revealed the most frequent morphological changes observed to be in the form of hydropic, microvacuolar, and macrovacuolar modifications (Table 6). These include fatty changes pertaining to lipid metabolism, namely steatosis. In CTR, only one animal (12.5%) exhibited hydropic degeneration, characterized by cellular swelling and cytoplasm with irregular areas lacking color. The other animals in this group displayed microvacuolar and macrovacuolar fatty changes. In the microvacuolar form, small vacuoles filled the cytoplasm without displacing the nucleus, whereas in macrovacuolar changes, the vacuoles were larger, and the nucleus was pushed towards the periphery (Figure 1). It is noteworthy that all these changes were confined to a very restricted area of the hepatic parenchyma, specifically the periportal zone (Figure 1D). In WD, five animals (55.6%) showed both microvacuolar and macrovacuolar changes simultaneously. In most of these animals, the alterations were distributed diffusely, observed throughout the entire hepatic lobule. Among the animals treated with GLE, both forms of changes were frequently present, with the diffuse distribution pattern being predominant (Figure 1A–C,E,F).

Regarding other hepatic alterations, focal chronic hepatitis was observed in one animal from CTR (12.5%), characterized by an inflammatory infiltrate predominantly consisting of lymphocytes and few macrophages. This was also seen in two animals from WD (22.2%) and in two animals from the GLE supplementation groups (one animal from WD+0.7% GLE (11.1%) and one from WD+1.4% GLE (10%)). Additionally, one animal in WD (11.1%) and two animals in WD+2.8% GLE (18.2%) exhibited multifocal hepatitis, with the same type of inflammatory infiltrate (Figure 1C–F).

Hepatic DNA damage and antioxidant enzyme activities are summarized in Table 7. The WD group had a higher GDI than CTR and all GLE-supplemented groups (*p* < 0.05). WD+2.8% GLE had the lowest GDI and differed significantly from all other groups (*p* < 0.05). Nonetheless, activities of antioxidant enzymes, including catalase (CAT) and glutathione reductase (GR), did not differ significantly between groups. Although the lowest mean activities were consistently observed in the WD group, these differences did not reach statistical significance (*p* > 0.05).

### 3.5. Adipose Tissue Phenotype

Histological evaluation of adipose tissue depots revealed depot-specific heterogeneity in adipocyte morphology (Table 8). WAT predominated in most depots; however, MLAs with a beige-like appearance and the co-occurrence of WAT and MLAs were also observed. This classification is descriptive and does not demonstrate functional adipose tissue browning.

**Figure 2:**
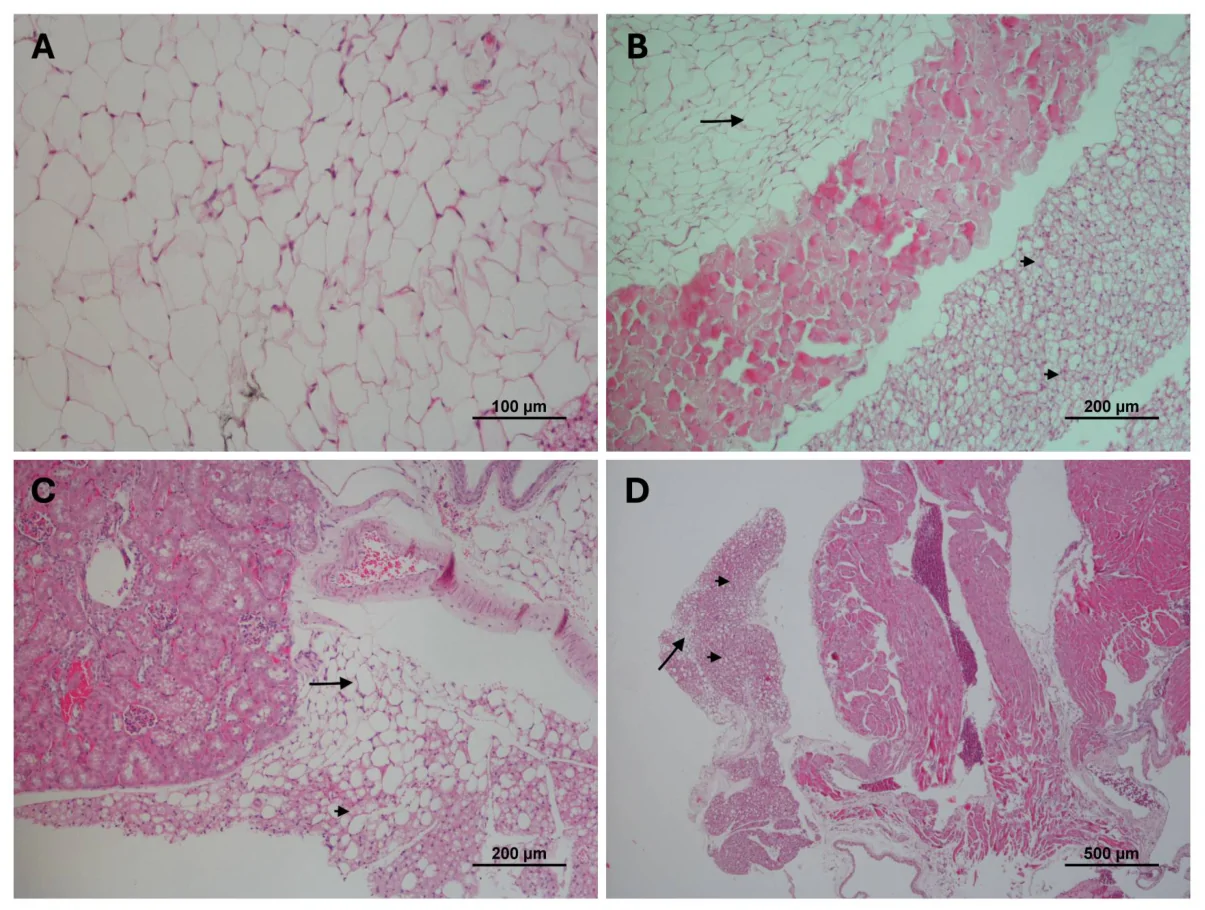
Representative histological micrographs of the different types of adipose tissue morphology: (**A**) white adipose tissue (WAT) from the abdomino-pelvic depot; (**B**) WAT (arrow) containing multilocular adipocytes (MLAs) with a beige-like appearance (arrowhead) in the anterior subcutaneous depot; (**C**) perirenal and (**D**) pericardial adipose tissue, where the presence of WAT and MLAs can be seen. Images illustrate the range of morphological patterns identified during histopathological evaluation and not specific experimental groups. Quantitative data are presented in Table 8.

In the abdomino-pelvic reserve (Figure 2A), WAT was predominant across all groups, being present in more than 60% of animals per group. MLAs with a beige-like appearance were detected in six animals from the non-supplemented groups (three in CTR and three in WD), while they were more frequent in the GLE-supplemented groups (7–9 animals), with a maximum incidence of 90% in WD+1.4% GLE. In the anterior subcutaneous depot, MLAs were the predominant morphological pattern in most groups, especially those supplemented with the GLE. MLAs were found alone in 100% of animals from WD+0.7% GLE and in only 50% of CTR animals (*p* < 0.05). WAT was detected less frequently in this depot, with a maximum of 25% in CTR animals. Simultaneous WAT and MLA presence (Figure 2B) was absent only in the WD+0.7% GLE group. In the posterior subcutaneous reserve, WAT was the dominant tissue in all groups, with values ranging from 88.9% (in WD+0.7% GLE) to 100% (in CTR and WD). MLAs were not found alone in any group, but the co-occurrence of WAT and MLAs was noted in only three animals, all from the GLE-supplemented groups (one per group). For visceral adipose tissue, particularly perirenal and pericardial adipose tissue (Figure 2C,D), WAT was again the most frequent tissue type, observed in over 60% of animals in each group, except WD+1.4% GLE and WD+2.8% GLE. Conversely, the co-occurrence of WAT and MLAs was found to be highest in the same groups (the two highest GLE concentrations), and differed significantly from CTR and WD+0.7% GLE (*p* < 0.05).

### 3.6. Renal Tissue Morphology

**Figure 3:**
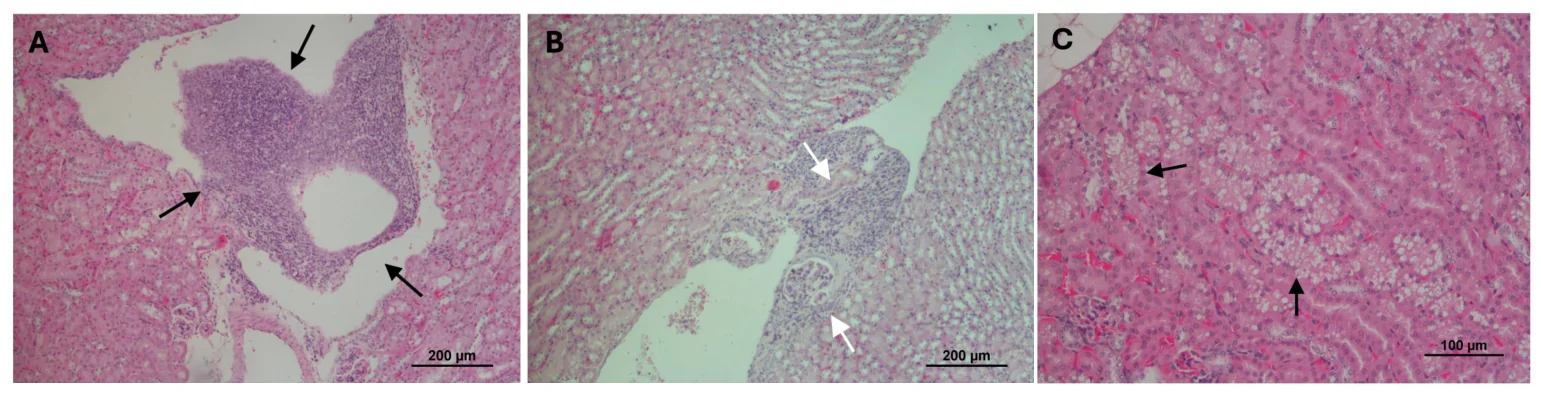
Representative micrographs of the histological changes found in the kidney: (**A**) chronic pyelonephritis (black arrows); (**B**) chronic interstitial nephritis with lymphoplasmocytic infiltrate around the corpuscles and convoluted tubes (white arrows); and (**C**) vacuolar changes in the distal convoluted tubes of the superficial cortex (black arrows). Images illustrate the range of lesions identified during histopathological evaluation and not specific experimental groups. Quantitative data of the inflammatory lesions are presented in Table 9.

Histological alterations in renal tissue were infrequent across experimental groups and were primarily inflammatory in nature (Table 9, Figure 3). When present, these changes were characterized by inflammatory infiltrates composed predominantly of lymphocytes, plasma cells, and macrophages, with lymphocytes being the predominant cell type. In the CTR group, only three out of eight animals (37.5%) presented normal renal histology. Chronic pyelonephritis was observed in another three animals (37.5%), while one animal (12.5%) exhibited chronic interstitial nephritis and one (12.5%) showed signs of pyelonephritis and inflammatory infiltrate around the arcuate arteries (vascular cuffs). The WD group had a higher proportion of normal kidneys (five out of nine animals, 55.6%) compared to CTR. However, histological changes were still present: one animal (11.1%) exhibited chronic interstitial nephritis, one (11.1%) had chronic pyelonephritis, and two animals (22.2%) showed pyelonephritis with vascular cuffs. In contrast, GLE-supplemented groups displayed fewer histological changes in comparison with the CTR and WD groups (*p* < 0.05). The WD+0.7% GLE group had eight out of nine animals (88.9%) with normal kidneys and only one case (11.1%) of pyelonephritis with vascular abnormalities. The WD+1.4% GLE group showed no histological alterations, with all 10 animals (100%) presenting normal renal tissue. Similarly, in the WD+2.8% GLE group, 10 out of 11 animals (90.9%) had normal kidneys, and only one animal (11.1%) displayed chronic interstitial nephritis.

## 4. Discussion

This exploratory study was conducted using WD-fed male C57BL/6J mice to reflect important features of diet-induced tissue-level alterations under controlled experimental conditions. In contemporary human populations, dietary patterns characterized by high consumption of energy-dense, ultra-processed foods and low adherence to healthy dietary habits have been consistently associated with metabolic dysregulation and systemic physiological alterations [41]. These observations reinforce the relevance of experimental models that reproduce Western-style diet consumption when investigating its physiological consequences. Compared with CTR, WD produced a limited set of statistically significant changes: lower relative right kidney weight, a shift in hepatic vacuolar changes from a predominantly localized periportal distribution to a diffuse distribution, and a higher hepatic GDI. In contrast, final body mass, Lee index, adiposity index, glucose-tolerance AUC, relative liver weight, and hepatic CAT and GR activities did not differ significantly between CTR and WD. Thus, under the present dietary conditions and study duration, the WD induced selected tissue-level alterations rather than a uniform systemic metabolic phenotype. While genetically modified mouse models offer experimental uniformity due to consistent phenotypes, they represent monogenic conditions that are not representative of the complete spectrum of human metabolic disorders; in contrast, diet-induced models integrate multiple factors, including nutrient composition, energy excess, lifestyle-related influences and tissue-specific susceptibility, thereby better capturing aspects of the multifactorial and heterogeneous nature of metabolic stress which may emerge at different rates and with variable magnitude [42,43]. Consequently, the interpretation of the present findings considers the integrated pattern of physiological responses rather than isolated parameters.

To address whether a dietary intervention could modulate these diet-induced metabolic alterations, WD-fed male C57BL/6J mice were supplemented with nutritionally realistic concentrations (0.7–2.8%, *w*/*w*) of a *G. lucidum* extract. The dietary inclusion levels were designed to mimic subchronic, consistent intake of a concentrated mushroom-derived ingredient rather than acute or pharmacological dosing. GLE supplementation was associated with specific tissue-level differences, where all GLE groups had lower hepatic GDI than WD and relative right kidney weights comparable to CTR. WD+1.4% GLE showed a higher frequency of localized periportal hepatic changes than WD, whereas the other GLE groups did not. WD+2.8% GLE also had lower relative liver weight than WD. GLE did not significantly affect final body mass, adiposity, glucose-tolerance AUC, or hepatic CAT and GR activities. The results therefore support modest, endpoint-specific associations rather than a generalized metabolic benefit or a defined mechanism of action. In this study, a standard chow-fed group receiving GLE was not included given the extensive body of literature documenting the toxicological safety and tolerability of *G. lucidum* extracts under normal physiological conditions [44,45], allowing the experimental design to focus specifically on the capacity of dietary GLE to modulate WD-induced tissue-level alterations while limiting the number of animals used.

Within this context, GLE supplementation significantly improved basal glycemic control, as reflected by a dose-dependent reduction in fasting blood glucose, despite the absence of significant effects on glucose tolerance. Fasting glucose and the glucose tolerance test provided different information in the present study. Fasting glucose was lower in WD+2.8% GLE than in CTR, but none of the GLE groups differed significantly from WD, and glucose tolerance was unchanged at both assessments. Fasting glucose reflects the basal glycemic state after food withdrawal and is an early indicator of diet-induced metabolic dysfunction and largely reflects hepatic insulin resistance and dysregulated gluconeogenesis [46], whereas the intraperitoneal glucose tolerance test assesses the integrated handling of an acute glucose load. The dissociation observed between fasting glycemia and glucose tolerance suggests that dietary GLE may have influenced basal hepatic glucose handling rather than peripheral glucose uptake. However, as insulin levels and molecular markers of hepatic glucose metabolism were not evaluated in the present study, this interpretation should be considered with caution and limited to the physiological observations obtained. While improvements in fasting glycemia following *G. lucidum* supplementation have been reported in experimental models [26,47], the present findings demonstrate that such effects may occur under conditions of subchronic dietary intake, independently of changes in body weight or glucose tolerance.

Given the central role of the liver in obesity-related metabolic stress [48,49], in this study WD-induced hepatic alterations were also studied. WD differed from CTR in the distribution of vacuolar changes and in GDI, but not in relative liver weight. The shift from localized periportal to diffuse vacuolar involvement indicates a broader anatomical extent of hepatocellular alteration with WD. However, H&E morphology alone cannot confirm that all vacuoles represent lipids or quantify hepatic lipid content. Among the GLE groups, WD+1.4% GLE showed a significant increase in localized periportal changes relative to WD, which may reflect changes in hepatic lipid handling [50], although this was not directly assessed. These findings align with previous reports indicating that *Ganoderma*-derived compounds modulate hepatic lipid metabolism in high-fat diet-fed mice [51,52]. Notably, these hepatic improvements occurred in parallel with a reduction in fasting glycemia, which is relevant given the liver’s importance in regulating glucose homeostasis [53]. Beyond lipid-related changes, dietary GLE supplementation also significantly reduced hepatic DNA damage, a known consequence of WD-induced metabolic dysfunction in the liver [54,55,56]. This reduction is especially meaningful in light of the involvement of genomic instability in the progression of obesity-associated liver pathology [57]. While *Ganoderma*-derived compounds have shown antigenotoxic activity in simplified assays [58], the present findings provide, to the best of our knowledge, the first evidence that subchronic dietary supplementation with *G. lucidum* reduces hepatic DNA damage under obesogenic conditions, despite unchanged hepatic CAT and GR activities. Although CAT and GR activities did not differ significantly between groups, both enzymes showed slightly higher values in the GLE-supplemented animals, a non-significant trend that may reflect a subtle enhancement of hepatic antioxidant defenses. In this context, the reduction in DNA damage observed in the highest GLE group may result from mechanisms not captured by the antioxidant enzymes assessed, including indirect effects related to metabolic regulation or cellular stress responses.

GLE supplementation was also found to influence depot-specific remodeling of the adipose tissue phenotype. MLAs with a beige-like appearance were more frequent in selected subcutaneous and visceral comparisons with GLE supplementation, whereas total adiposity was unchanged. Such phenotypic remodeling is increasingly recognized as an adaptive response to adjust energy handling and metabolic capacity [59,60], which has been associated with lipid partitioning, adipose tissue–liver crosstalk that limits hepatic lipid deposition [61,62] and contributes to improved basal metabolic regulation [63]. Multilocular morphology is compatible with a beige-like phenotype but is not sufficient to demonstrate adipose tissue browning or increased thermogenic function. However, this interpretation remains preliminary and would benefit from confirmation through targeted molecular and functional analyses of adipose tissue activity. While previous studies with *G. lucidum* have largely focused on adipose tissue accumulation [64,65,66], evidence supporting its remodeling toward metabolically active phenotypes remains limited. Nevertheless, confirmation through targeted analyses of thermogenic and metabolic biomarkers (UCP1, PGC1α and PRDM16) is required to determine whether the observed phenotype reflects functional browning.

Renal histological alterations were infrequent across experimental groups and did not indicate kidney injury. Importantly, dietary GLE supplementation was not associated with increased renal inflammation or structural alterations, supporting its renal tolerability. Additionally, the lower relative right kidney weight observed in WD was not present in the GLE groups, which had values comparable to CTR. Nevertheless, renal function biomarkers were not measured, and the low absolute lesion frequencies limit mechanistic interpretation. The absence of a dose-related increase in renal lesions provides no indication of renal histological toxicity attributable to GLE under the conditions tested, in agreement with previous reports on these extracts [67,68].

Given the exploratory nature of this preclinical study, a few limitations should be acknowledged when interpreting the results. The analysis of metabolic endpoints such as insulin levels, insulin resistance indices, lipid profiles, and markers of hepatic metabolism or inflammation would greatly improve the interpretation of the metabolic effects. Hepatic vacuolar changes were evaluated using H&E staining without lipid-specific confirmation or morphometric quantification. The adipose tissue classification was based solely on morphology and was not validated using thermogenic markers or functional assays. Furthermore, while the sample size is consistent with commonly used experimental designs in diet-induced metabolic studies and allows the detection of biologically relevant effects, variability inherent to complex metabolic models should be considered when interpreting individual parameters. Future studies should focus on clarifying the molecular mechanisms underlying the tissue-specific metabolic responses observed, particularly through integrated molecular analyses of hepatic and adipose tissue signaling pathways, as well as more comprehensive metabolic profiling. Finally, assessing the bioavailability and systemic distribution of *G. lucidum* bioactives following dietary intake would further strengthen interpretation of these findings.

## 5. Conclusions

This exploratory study indicates that dietary GLE was associated with selected tissue-specific differences in WD-fed mice, most consistently lower hepatic DNA damage and depot-specific variation in multilocular adipocyte morphology. These observations were not accompanied by significant changes in final body mass, adiposity, glucose-tolerance AUC, or hepatic CAT and GR activities. The findings therefore support hypothesis generation regarding hepatic genomic integrity and adipose tissue morphology, highlighting the need for further mechanistic studies.

## Figures and Tables

**Table 1:** Initial and final food and water intake in all experimental groups. Data are presented as mean ± SEM.

Group	Food (g)		Water (g)	
	Initial	Final	Initial	Final
CTR	4.32 ± 0.20	2.49 ± 0.04	3.83 ± 0.87	4.47 ± 0.94
WD	4.37 ± 0.62	3.77 ± 0.51	3.61 ± 0.61	4.14 ± 0.03
WD+0.7% GLE	2.98 ± 0.36	4.12 ± 0.78	3.43 ± 0.68	2.93 ± 0.14
WD+1.4% GLE	3.70 ± 0.29	4.29 ± 0.93	4.56 ± 0.51	3.81 ± 0.57
WD+2.8% GLE	3.55 ± 0.30	3.41 ± 0.54	3.67 ± 0.20	4.38 ± 0.17

No statistically significant differences were observed between groups at either time point (*p* > 0.05). CTR: Western control diet; WD: Western diet with 0.2% cholesterol; GLE: *Ganoderma lucidum* extract.

**Table 2:** Initial and final body mass, Lee index and adiposity index in all experimental groups. Data are presented as mean ± SEM.

Group	Body Mass (g)		Lee Index (g cm^−1^)	Adiposity Index (%)
	Initial	Final		
CTR (*n* = 8)	27.12 ± 0.56 ^b^	32.18 ± 1.04 ^a^	332.03 ± 12.43 ^a^	7.64 ± 0.51 ^a^
WD (*n* = 9)	29.97 ± 0.53 ^a^	34.92 ± 0.79 ^a^	334.62 ± 3.13 ^a^	7.86 ± 0.58 ^a^
WD+0.7% GLE (*n* = 9)	28.34 ± 0.58 ^ab^	32.17 ± 1.16 ^a^	344.74 ± 3.36 ^a^	8.57 ± 0.75 ^a^
WD+1.4% GLE (*n* = 10)	29.51 ± 0.69 ^a^	33.04 ± 0.84 ^a^	344.85 ± 2.23 ^a^	8.18 ± 0.36 ^a^
WD+2.8% GLE (*n* = 11)	29.90 ± 0.27 ^a^	34.27 ± 0.80 ^a^	351.77 ± 3.69 ^a^	9.41 ± 0.58 ^a^

Different superscript letters within the same column indicate statistically significant differences between groups (*p* < 0.05). CTR: Western control diet; WD: Western diet with 0.2% cholesterol; GLE: *Ganoderma lucidum* extract.

**Table 3:** Glycemic parameters (glucose tolerance assessment and fasting glucose at sacrifice) in Western diet-fed mice supplemented with GLE. Data are presented as mean ± SEM.

Group	Glucose Tolerance (AUC, mg·min·dL^−1^)		Fasting Glucose at Sacrifice (mg dL^−1^)
	Week 7	Week 13	
CTR (*n* = 8)	28,557 ± 826 ^a^	29,009 ± 1832 ^a^	371 ± 21 ^a^
WD (*n* = 9)	28,806 ± 1273 ^a^	32,604 ± 4373 ^a^	312 ± 22 ^ab^
WD+0.7% GLE (*n* = 9)	28,868 ± 762 ^a^	31,178 ± 3699 ^a^	339 ± 23 ^ab^
WD+1.4% GLE (*n* = 10)	30,696 ± 1943 ^a^	30,392 ± 1545 ^a^	297 ± 25 ^ab^
WD+2.8% GLE (*n* = 11)	28,305 ± 1017 ^a^	33,113 ± 4208 ^a^	260 ± 16 ^b^

Different superscript letters within the same column indicate statistically significant differences between groups (*p* < 0.05). CTR: Western control diet; WD: Western diet with 0.2% cholesterol; GLE: *Ganoderma lucidum* extract. For glucose tolerance, serum glucose (mg dL^−1^) was measured immediately before and at 30, 60, and 120 min after injection, with the area under the curve (AUC; mg × min dL^−1^) then being calculated and glucose concentration expressed as a function of time.

**Table 4:** Relative organ weights in all experimental groups. Data are presented as mean ± SEM.

Organ	Relative Organ Mass (g Organ/g Body Weight)				
	CTR (*n* = 8)	WD (*n* = 9)	WD+0.7% GLE (*n* = 9)	WD+1.4% GLE (*n* = 10)	WD+2.8% GLE (*n* = 11)
Heart	0.005 ± 0.0003 ^ab^	0.006 ± 0.0003 ^ab^	0.005 ± 0.0003 ^ab^	0.006 ± 0.0002 ^a^	0.005 ± 0.0002 ^b^
Right kidney	0.008 ± 0.0002 ^a^	0.007 ± 0.0002 ^b^	0.008 ± 0.0002 ^a^	0.008 ± 0.0002 ^a^	0.008 ± 0.0002 ^a^
Left kidney	0.008 ± 0.0002 ^a^	0.008 ± 0.0001 ^a^	0.007 ± 0.0002 ^b^	0.008 ± 0.0001 ^a^	0.008 ± 0.0003 ^a^
Liver	0.046 ± 0.003 ^ab^	0.047 ± 0.004 ^a^	0.046 ± 0.002 ^ab^	0.040 ± 0.001 ^ab^	0.038 ± 0.001 ^b^
Lungs	0.006 ± 0.0002 ^a^	0.006 ± 0.0002 ^a^	0.006 ± 0.0002 ^a^	0.006 ± 0.0003 ^a^	0.005 ± 0.0002 ^b^
Spleen	0.003 ± 0.0004 ^a^	0.003 ± 0.0002 ^a^	0.003 ± 0.0001 ^a^	0.003 ± 0.0001 ^a^	0.003 ± 0.0001 ^a^
Thymus	0.001 ± 0.0001 ^a^	0.001 ± 0.0001 ^a^	0.001 ± 0.0002 ^a^	0.001 ± 0.0001 ^a^	0.001 ± 0.0001 ^a^

Different superscript letters within the same row indicate statistically significant differences between groups (*p* < 0.05). CTR: Western control diet; WD: Western diet with 0.2% cholesterol; GLE: *Ganoderma lucidum* extract.

**Table 5:** Relative weights of adipose tissue depots in all experimental groups. Data are presented as mean ± SEM.

Group	Relative Adipose Tissue Mass (g AT Depot/g Body Weight)		
	Abdomino-Pelvic AT	Anterior Subcutaneous AT	Posterior Subcutaneous AT
CTR (*n* = 8)	0.036 ± 0.003 ^a^	0.022 ± 0.002 ^a^	0.019 ± 0.001 ^ab^
WD (*n* = 9)	0.040 ± 0.003 ^a^	0.022 ± 0.002 ^a^	0.017 ± 0.002 ^b^
WD+0.7% GLE (*n* = 9)	0.038 ± 0.004 ^a^	0.027 ± 0.002 ^a^	0.021 ± 0.002 ^ab^
WD+1.4% GLE (*n* = 10)	0.036 ± 0.002 ^a^	0.025 ± 0.001 ^a^	0.020 ± 0.001 ^ab^
WD+2.8% GLE (*n* = 11)	0.047 ± 0.003 ^a^	0.024 ± 0.002 ^a^	0.023 ± 0.001 ^a^

Different superscript letters within the same column indicate statistically significant differences between groups (*p* < 0.05). CTR: Western control diet; WD: Western diet with 0.2% cholesterol; GLE: *Ganoderma lucidum* extract; AT: adipose tissue.

**Table 6:** Absolute (and relative, %) frequencies of hepatic histological changes in all experimental groups.

Group	Histological Changes (Degeneration)			Distribution	
	Hydropic	Microvacuolar	Microvacuolar and Macrovacuolar	Diffused	Localized (Periportal)
CTR (*n* = 8)	1 ^a^ (12.5%)	7 ^a^ (87.5%)	5 ^a^ (62.5%)	0 ^a^ (0.0%)	7 ^a^ (87.5%)
WD (*n* = 9)	1 ^a^ (11.1%)	5 ^a^ (55.6%)	5 ^a^ (55.6%)	5 ^b^ (55.6%)	1 ^b^ (11.1%)
WD+0.7% GLE (*n* = 9)	0 ^a^ (0.0%)	8 ^a^ (88.9%)	8 ^ab^ (88.9%)	5 ^b^ (55.6%)	3 ^bc^ (33.3%)
WD+1.4% GLE (*n* = 10)	0 ^a^ (0.0%)	10 ^a^ (100%)	10 ^b^ (100.0%)	4 ^b^ (40.0%)	6 ^ac^ (60.0%)
WD+2.8% GLE (*n* = 11)	0 ^a^ (0.0%)	9 ^a^ (81.8%)	9 ^ab^ (81.8%)	7 ^b^ (63.6%)	2 ^b^ (18.2%)

Different superscript letters within the same column indicate statistically significant differences between groups (*p* < 0.05). CTR: Western control diet; WD: Western diet with 0.2% cholesterol; GLE: *Ganoderma lucidum* extract.

**Table 7:** Genetic damage index [arbitrary units (AU) of DNA breaks] and oxidative stress markers [catalase (CAT) and glutathione reductase (GR)] in the liver in all experimental groups. Data are presented as mean ± SEM.

Group	GDI (AU)	Antioxidant Enzymes	
		CAT (mmol H_2_O_2_ min^−1^ mg^−1^ Protein)	GR (μM NADPH Oxidized min^−1^ mg^−1^ Protein)
CTR (*n* = 8)	144.36 ± 1.19 ^b^	441.18 ± 57.80 ^a^	0.284 ± 0.030 ^a^
WD (*n* = 9)	157.23 ± 1.22 ^a^	430.29 ± 68.74 ^a^	0.278 ± 0.007 ^a^
WD+0.7% GLE (*n* = 9)	141.22 ± 0.81 ^b^	446.95 ± 75.65 ^a^	0.309 ± 0.073 ^a^
WD+1.4% GLE (*n* = 10)	143.00 ± 1.11 ^b^	461.42 ± 53.70 ^a^	0.324 ± 0.017 ^a^
WD+2.8% GLE (*n* = 11)	136.33 ± 1.03 ^c^	496.19 ± 55.01 ^a^	0.334 ± 0.013 ^a^

Different superscript letters within the same column indicate statistically significant differences between groups (*p* < 0.05). CTR: Western control diet; WD: Western diet + 0.2% cholesterol; GLE: *Ganoderma lucidum* extract.

**Table 8:** Absolute (and relative, %) frequencies of white adipose tissue (WAT), multilocular adipocytes (MLAs) with a beige-like appearance, and their co-occurrence in each adipose depot across experimental groups.

Group	Abdomino-Pelvic AT		Anterior Subcutaneous AT			Posterior Subcutaneous AT		Visceral AT	
	WAT	WAT+MLAs	MLAs	WAT	WAT+MLAs	WAT	WAT+MLAs	WAT	WAT+MLAs
CTR (*n* = 8)	5 ^a^ (62.5%)	3 ^a^ (37.5%)	4 ^a^ (50.0%)	2 ^a^ (25.0%)	2 ^a^ (25.0%)	8 ^a^ (100.0%)	0 ^a^ (0.0%)	5 ^a^ (62.5%)	3 ^a^ (37.5%)
WD (*n* = 9)	6 ^a^ (66.7%)	3 ^a^ (33.3%)	6 ^ab^ (66.7%)	1 ^a^ (11.1%)	2 ^a^ (22.2%)	9 ^a^ (100.0%)	0 ^a^ (0.0%)	8 ^ab^ (88.9%)	1 ^ab^ (11.1%)
WD+0.7% GLE (*n* = 9)	7 ^a^ (77.8%)	2 ^a^ (22.2%)	9 ^b^ (100.0%)	0 ^a^ (0.0%)	0 ^a^ (0.0%)	8 ^a^ (88.9%)	1 ^a^ (11.1%)	7 ^b^ (77.8%)	2 ^b^ (22.2%)
WD+1.4% GLE (*n* = 10)	9 ^a^ (90.0%)	1 ^a^ (10.0%)	9 ^ab^ (90.0%)	0 ^a^ (0.0%)	1 ^a^ (10.0%)	9 ^a^ (90.0%)	1 ^a^ (10.0%)	3 ^c^ (30.0%)	7 ^c^ (70.0%)
WD+2.8% GLE (*n* = 11)	7 ^a^ (63.6%)	4 ^a^ (36.4%)	9 ^ab^ (81.8%)	0 ^a^ (0.0%)	2 ^a^ (18.2%)	10 ^a^ (90.9%)	1 ^a^ (9.1%)	4 ^bc^ (36.4%)	7 ^bc^ (63.6%)

Different superscript letters within the same column indicate statistically significant differences between groups (*p* < 0.05). CTR: Western control diet; WD: Western diet with 0.2% cholesterol; GLE: *Ganoderma lucidum* extract; AT: adipose tissue; MLAs: multilocular adipocytes; WAT: white adipose tissue.

**Table 9:** Absolute (and relative, %) frequencies of renal histological changes.

Group	Histological Changes		
	Chronic Interstitial Nephritis	Chronic Pyelonephritis	Pyelonephritis + Vascular Cuffs
CTR (*n* = 8)	1 ^a^ (12.5%)	3 ^a^ (37.5%)	1 ^a^ (12.5%)
WD (*n* = 9)	1 ^a^ (11.1%)	1 ^ab^ (11.1%)	2 ^a^ (22.2%)
WD+0.7% GLE (*n* = 9)	0 ^a^ (0%)	0 ^b^ (0%)	1 ^a^ (11.1%)
WD+1.4% GLE (*n* = 10)	0 ^a^ (0%)	0 ^b^ (0%)	0 ^a^ (0%)
WD+2.8% GLE (*n* = 11)	1 ^a^ (11.1%)	0 ^b^ (0%)	0 ^a^ (0%)

Different superscript letters within the same column indicate statistically significant differences between groups (*p* < 0.05). CTR: Western control diet; WD: Western diet with 0.2% cholesterol; GLE: *Ganoderma lucidum* extract.

## Data Availability

The original contributions presented in this study are included in the article. Further inquiries can be directed to the corresponding authors.

## Abbreviations

The following abbreviations are used in this manuscript:

AT	Adipose tissue
AUC	Area under the curve
CAT	Catalase
CTR	Western diet control group
GDI	Genetic damage index
GLE	*Ganoderma lucidum* extract
GR	Glutathione reductase
H&E	Hematoxylin–eosin
MLAs	Multilocular adipocytes
SEM	Standard error of the mean
WD	Western diet
WAT	White adipose tissue
